# Deep Neural Architectures for Contrast Enhanced Ultrasound (CEUS) Focal Liver Lesions Automated Diagnosis †

**DOI:** 10.3390/s21124126

**Published:** 2021-06-16

**Authors:** Cătălin Daniel Căleanu, Cristina Laura Sîrbu, Georgiana Simion

**Affiliations:** Applied Electronics Department, Faculty of Electronics, Telecommunications and Information Technologies, Politehnica University Timișoara, 300223 Timișoara, Romania; catalin.caleanu@upt.ro (C.D.C.); cristina.sirbu@upt.ro (C.L.S.)

**Keywords:** contrast enhanced ultrasound imaging, CEUS, focal liver lesions, FLL, deep learning, deep neural networks

## Abstract

Computer vision, biomedical image processing and deep learning are related fields with a tremendous impact on the interpretation of medical images today. Among biomedical image sensing modalities, ultrasound (US) is one of the most widely used in practice, since it is noninvasive, accessible, and cheap. Its main drawback, compared to other imaging modalities, like computed tomography (CT) or magnetic resonance imaging (MRI), consists of the increased dependence on the human operator. One important step toward reducing this dependence is the implementation of a computer-aided diagnosis (CAD) system for US imaging. The aim of the paper is to examine the application of contrast enhanced ultrasound imaging (CEUS) to the problem of automated focal liver lesion (FLL) diagnosis using deep neural networks (DNN). Custom DNN designs are compared with state-of-the-art architectures, either pre-trained or trained from scratch. Our work improves on and broadens previous work in the field in several aspects, e.g., a novel leave-one-patient-out evaluation procedure, which further enabled us to formulate a hard-voting classification scheme. We show the effectiveness of our models, i.e., 88% accuracy reported against a higher number of liver lesion types: hepatocellular carcinomas (HCC), hypervascular metastases (HYPERM), hypovascular metastases (HYPOM), hemangiomas (HEM), and focal nodular hyperplasia (FNH).

## 1. Introduction

Computer-aided diagnosis (CAD) has been applied to address several diagnostic problems of digital images obtained from different sensing modalities, starting from conventional projection radiography, and continuing with computed tomography (CT), magnetic resonance imaging (MRI), and ultrasound (US) imaging, e.g., detection and characterization of breast cancer lesions obtained from digital mammography [1], pulmonary diseases [2], colonography [3], or brain tumor detection [4]. Conventional ultrasound has been shown to be largely outperformed by contrast-enhanced CT or liver MRI studies. Intravenously injected contrast agents have been long used with CT and MR imaging modalities, to enhance visualization of microcirculation. One approach to make microcirculation detection easier with ultrasound is to introduce scatterers into the blood, in order to increase the backscatter signal. The scatterers have to be small enough to pass from the venous to the arterial side of circulation, in lungs. With the recent availability of second-generation contrast agents for US imagery, like Sono Vue (Bracco, Milan, Italy) or Sonazoid (Daiichi Sankyo, Tokyo, Japan), diagnosis of focal liver diseases in CEUS has been proved to become a reliable solution.

Our aim in this article is to develop a CAD system for automated diagnosis of FLLs from CEUS images. Its output is a decision for a certain diagnosis, but this decision is intended to be used only as a second opinion. A CAD system performs pattern recognition tasks, thus (1) image preprocessing, (2) definition and extraction of regions of interest (ROI), (3) feature extraction and selection and (4) classification stages should be implemented. The first block operates at pixel level. Its objective is to improve the image quality for further processing. One of the simplest types of preprocessing tasks is gray level remapping. Linear or nonlinear mappings allow gray level normalization of images obtained at different times with different devices and machine settings for subsequent comparison. Spatial or spatial-temporal filters can be further used to reduce the effects of image noise. Noise removal in US imagery needs special care, as these images are affected by speckle noise [5]. ROI definition is a particular type of image segmentation, where the objective is to extract only one region, which is of interest for further examination. The task is often very challenging, especially if done fully automatically. Methods used in medical image segmentation depend on applications. Different requirements are encountered in applications, depending on the imaging modality and the morphology of the targeted region. Among the most widely used approaches in automatic image segmentation are the mean shift [6], active contour-based segmentation [7] and tree based [8]. Feature extraction performs the most drastic reduction of data: from images to a small set of measurements made on the ROI. Features need de be defined such that they contain all the information needed for detection and accurate estimation of pathological aspects, leading to best medical decisions. Based on the available learning feature vectors from known classes and the feature vector extracted from the current image, the last block of the CAD system classifies this vector into one of the predefined classes. Although the design of a good pattern classifier is less application-dependent than the previous three processing stages, there is no simple way to predict which type of classifier will perform best for a particular application. For the classification stage, the kNN is one of the most effective solution in many applications. Support vector machines (SVM) are known for their ability to generalize well from a scarce training set and much research effort was directed to ensemble classifiers, like bagging, boosting and decision trees [9].

In our method, the last two steps are fused. By employing a deep learning (DL) approach, the features are automatically learnt from data and further classified by the fully connected upper layer. More exactly, we extend our previous work [10] as follows:

(1) We introduce a new, more realistic, evaluation procedure, referred to as leave-one-patient-out (LOPO). To our best knowledge, this is the single CAD for CEUS FLLs work in which the evaluation does not follow the standard training/validation/testing split applied with respect to *images* [11,12,13,14]. The main drawback of the latter approach is that the images from the training and testing sets, obviously distinct, may still origin from the same patient, thus making the evaluation easier, and not suitable for claiming CAD in-field performances.
(2) The above-mentioned procedure enabled us to define and implement different voting schemes for *patient-oriented* lesion diagnosis. For example, a hard vote scheme uses predicted class labels for majority rule voting, whereas soft voting predicts the class label based on the argmax of the sums of the predicted probabilities.
(3) Our early work was the first one which uses custom designed 2D-DCNN for implementing an automated CAD for CEUS FLLs. In the current work, we further extend the study by employing modern DNN architectures available through Keras Applications. They are deep learning models that are made available alongside pre-trained weights used in this paper in various forms (transfer learning/feature extraction, fine-tuning or train from scratch). In our study, a special emphasis is put on TinyML/small memory footprint models, as we intend to transfer the CAD into a medical embedded system.

## 2. State of the Art

On one hand, US is the most performed imaging investigation in clinical practice. CEUS imaging is an improved ultrasound-based technology having a superior sensitivity, compared to that of CT or MRI [15]. On the other hand, DL has recently emerged as the leading machine learning tool in various research fields. It is one of the most popular artificial intelligence techniques used in the medical field, especially for image and video processing, thus numerous studies applying deep learning to ultrasound/CEUS imaging have been actively conducted [16,17]. The current section reviews CEUS based DL approaches for medical investigation, in general, and for FLL diagnosis, in particular.

### 2.1. Deep Learning Based CEUS for Medical Investigations

Being a non-invasive, less time consuming and relatively cheap procedure, nowadays CEUS investigation is used for various pathologies ranging from liver lesions [18], thyroid nodules [19,20] prostate cancer [21,22], rectal cancer [23,24] breast cancer [25,26] to kidney cystic lesions or tumors, [27,28]. In order to diagnose these pathologies from CEUS imaging, different methods can be used, but only few approaches for some pathologies take advantage of DNN and its associate learning paradigm DL, as follows.

Thyroid nodules diagnosis is performed in [20] on CEUS images using a hierarchical temporal attention network (HiTAN). The authors divided the algorithm in two categories: one is for the enhancement representation learning, and the other is for hierarchical lesion recognition. For enhancement learning, they are segmenting the frames using a CEUS-Net previously developed in [29]. The network consists of convolutional layers, local pooling layers and a final BatchNorm and ReLu activation function. In the hierarchical lesion recognition module, Gated Recurrent Units (GRUs) are employed to connect two consecutive classification tasks. The dataset consists of 325 patients with 336 lesions, including two types of benign nodules and two types of malign nodules. The experimental data shows an accuracy of 80.18%.

Prostate cancer is detected in [22] using three-dimensional convolution operation on CEUS images with deep neural networks. The framework extracts both spatial and temporal features. The convolutional neural network proposed consists of three types of layers, i.e., three convolutional layers, two sub-sampling pooling layers and one fully connected classification layer. The network was trained using stochastic diagonal Levenberg-Marquardt method. The training data set consists of 47,582 image samples, and the results show a high detection accuracy of over 90%.

In [24] rectal cancer is diagnosed. Feature extraction is performed using AlexNet, VGG16 and Resnet50, by combining and normalizing these features using a three layer fully connected neural network, they reach an accuracy of 87.91%

Yang et al. [25] combined B-mode ultrasound data and CEUS data using a temporal sequence dual-branch network to classify breast cancer. A ResNet18 network is used to extract spatial features from B-mode ultrasound video, and a 3D-based R(2 + 1)D network is used to extract temporal features from CEUS video. For the classification step, they propose to fuse these data using a temporal sequence regression mechanism, a loss to make the network pay more attention to the temporal information. They report a 4% higher accuracy than other state-of-art approaches in breast cancer classification.

### 2.2. Deep Learning Based CEUS for FLL Investigation

So far, we have mentioned only a few of the many works on computer aided diagnosis of lesions using DNN. Deep learning is a popular solution for detecting liver lesions in US [30,31] and currently is emerging as a promising solution for the automatic diagnosis during CEUS investigation. In [30] the authors train a residual network (ResNet) to to differentiate between malignant and benign focal solid liver lesions in abdominal ultrasound images. The model architecture is based on ResNet50, but the authors introduce several specific modifications. Among these, the SoftMax fully connected layer was replaced with a multi-layer perceptron with ReLU activation functions and a single output neuron with sigmoid activation function. The output neuron provides the probability that the lesion is malignant or benign. The dataset consists of 911 images of lesion from 596 patients. Out of the 911 images, 535 images contained malignant lesions and 376 images were benign lesions. The regions of interest were manually selected by a specialist. During training, the images were automatically augmented to increase the data set. The authors experiment with different combination of the dataset, and the highest accuracy achieved by the proposed model is 84%.

The work from [31] presents a classification framework that uses deep learning to diagnose three types of focal lesions: (Cyst, HEM, HCC). To remove unwanted artefacts and noise from the ultrasonic images the approach starts with a preprocessing stage that applies an anisotropic diffusion filter. The filter increases contrast and removes noise while keeping, or even enhancing edges. After the preprocessing stage, the ROI is extracted using a level set and fuzzy C-means clustering algorithm. A special type of feed forward ANN, called sparse auto-encoder (SAE), is used on the extracted ROIs for classification. One SAE contains an input layer, a hidden layer, and an output layer. The input and the output layer have the same size. Several SAEs are stacked to form the processing engine. Finally, the output of the stacked sparse auto-encoder is feed into a SoftMax classifier to determine the class of the lesions (Cyst, HEM, HCC and normal). The method was trained and tested on 110 US images and the authors show it has an accuracy of 97.2%.

A CAD system is presented in [11] where 3D-CNN are used to extract temporal and spatial features to detect FNH and HCC lesions. The training and detection are performed on 4420 samples, obtained from 242 tumors, with 2110 HCCs and 2310 FNHs lesions. Texture and edges from previous feature maps are integrated by the 2D convolutional kernel to extend the detection into a 3D space, therefore the temporal dimension is given by a sliding window of convolution. ReLU activation function is used and the 3D MaxPooling kernel was used after the convolution layer. Results show an accuracy of 93.1% for the proposed method.

In [14] Guo et al. apply deep canonical correlation analysis (DCCA) on pairs of CEUS images from different phases to extract features. Features are extracted from each phase (arterial, portal, late) and then pairs of features A-P, A-L, P-L are processed by the DCCA algorithm. These features are further classified using a multiple learning kernel classifier by discriminating benign liver tumor from malign liver cancers. Evaluation was performed on data from 93 patients, 46 which had benign tumors and 47 malignant cancers. A diagnosis accuracy of 90.41% is reported by the authors.

HCC tumors are also detected by Vancea et al. in [12] by employing deep learning techniques and CNN models. The authors trained and tested UNet, ERFNet and EDANet as they are suited for high quality segmentation. A dataset of 102 B-mode ultrasound images was used to train the networks. The Intersection over Union Metric (IoU) was used to evaluate the three network architectures and the authors concluded that ERFNet provides the best results with a IoU of 80.35%.

Time intensity curves (TIC) are representation of how contrast changes during CEUS investigation. Analyzing the TICs helps to determine whether the lesions are benign or malign. TICs are extracted from the CEUS frames using sparse non-negative matrix factorization which calculates the sparseness of each pixel based on the amount of mixing. The authors [32] train a deep belief network to analyze the TICs. DBN is a type of neural network formed of several layers of Boltzmann machines. The experimental results are generated on a dataset which contains 22 patients with 26 lesions. Out of 26 lesions, six are HCCs, 10 cavernous hemangiomas (CHs), four liver abscesses, three METAs and three localized fat springs (LFSs). By using deep learning to classify benign and malign focal liver lesions, from the extracted TICs, the authors achieved an accuracy of 86.36%.

TICs are also used as features in the work of Streba et al. [13]. These features are fed in an artificial neural network in order to classify the liver tumors with an accuracy of 87.12%. The experiments were performed on 112 patients which were randomly divided between training and testing data sets. The tumors are divided into five classes: HCC, hypervascular metastasis, hypovascular metastasis, HEM, fatty focal change.

## 3. Materials and Methods

### 3.1. Materials

The dataset used in this work was collected by the Department of Gastroenterology and Hepatology from ‘‘Victor Babes’’ University of Medicine and Pharmacy, Timisoara, Romania. All examinations were made with an Acuson S2000 ultrasound machine (Siemens, Berlin, Germany). From it we selected 91 patients. During the investigation, the probe of the ultrasound device is not in contact with the patient all the time; this is done in order to avoid the vanishing of echogenic gas bubbles from the injected agent needed for this type of procedure. The total number of processed video files is 273, for five types of liver lesions. The number of patients from each category is different, as can be seen in Table 1.

For each video file we have associated the coordinates of the ROIs. The ROIs were manually placed by experimented doctors. All examinations were made by experienced operators (Level II—advanced and III—expert, according to the Romanian Society of Ultrasound in Medicine and Biology classification). Each examination respected the standard of the 2012 European Federation of Societies for Ultrasound in Medicine and Biology (EFSUMB) guideline protocol for CEUS. According to [33], contrast enhanced CT, MRI, or histology were available in each case to confirm the final diagnosis.

The cases were manually selected from the provided video files according to certain quality parameters, e.g., the enhancing pattern, good ultrasound examination and good acoustic window. The number of samples was optimally determinate from (1) the lengths of the available video investigations and (2) the FPS acquisition rate.

We select 50 samples from each investigation phase, resulting in roughly 150 ROI images per patient. In the arterial phase, the sampling step is performed when the echogenicity starts to increase. The total number of acquired images is equal to 12,119. The size of these ROI images is varying.

ROI examples taken from each of the three following groups:

- Arterial (Beginning: 10–20 s, End: 25–35 s)
- Portal (Beginning: 30–45 s, End: 120 s)
- Late (Beginning: 120 s, End: until the disappearance of the bubbles)

**Figure 1:**
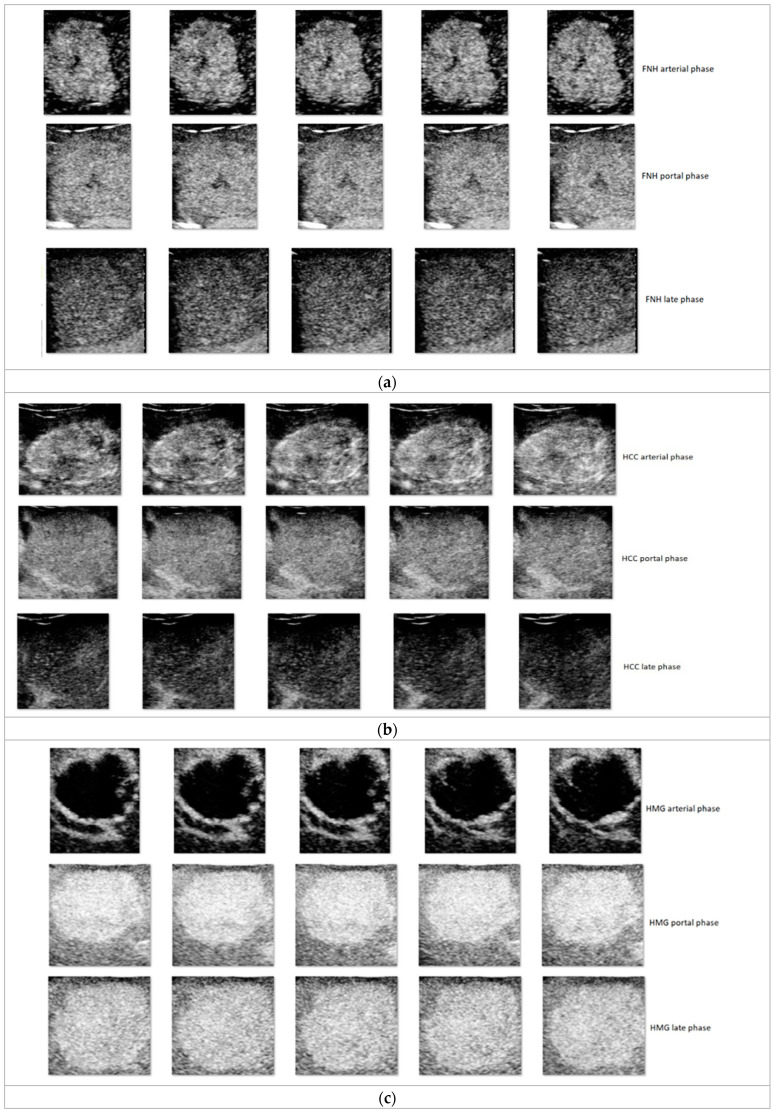

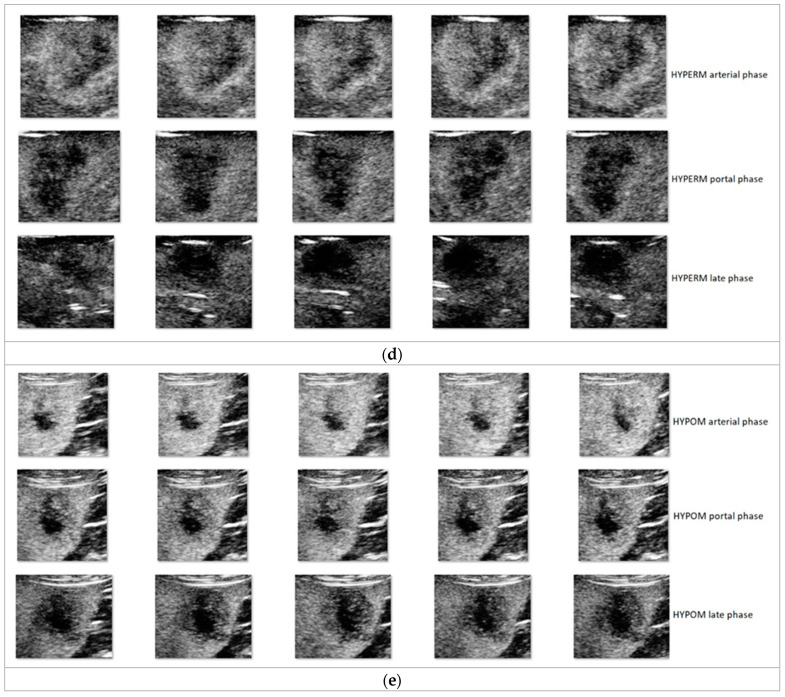
Five ROI representative examples, sampled at 10 frames difference, from each of the three (arterial, portal, late) CEUS exploration phases: (**a**) FNH; (**b**) HCC; (**c**) HMG; (**d**) HYPERM; (**e**) HYPOM.

CEUS exploration phases are depicted in Figure 1.

### 3.2. Method

**Figure 2:**
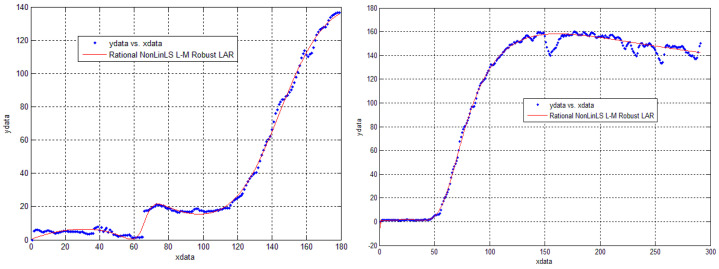
Curve Fitting Results. The algorithm used for calculating the polynomial coefficients was Levenberg-Marquardt. The best result was obtained for *m = n* = 5.

Many approaches use time intensity curves (TICs) to classify the liver lesions. That is why our earliest work [34] focused on TIC analysis in the arterial phase of CEUS investigations. The liver lesions were marked by doctors and we computed the mean of intensity through time, considering each frame from the arterial phase. These curves were affected by noise from several sources: the propagation of ultrasound waves through the soft tissue but also the lesion movement produced by patient heartbeat and the investigation probe. In order to reduce the influence of these aspects, a parametric curve-fitting, having a rational regression model:

(1) $$y=\frac{\sum_{i=1}^{n+1}p_{i}x^{n+1-i}}{x^{m}+\sum_{i=1}^{m}q_{i}x^{m-1}},$$

was further defined, with the smoothing effect (red line) shown in Figure 2.

For the fitted curve, the following parameters were extracted: rise time, settling times and peak time. Based on these features, we classified the liver lesions in four classes and obtained the following accuracy per class of: 37% for hepatocellular carcinoma, 82% for hemangioma, 65% for focal nodular hyperplasia and 72% for hypervascular metastasis; the overall classification rate was 64%. The dataset used in this work had 37 cases, 10 HCCs, 10 HEM, 10 FNH and seven HYPERM. A main drawback of this approach is that it took into consideration just the arterial phase, and no spatial information (lesion shape/pattern) was used to classify the liver lesions.

**Figure 3:**
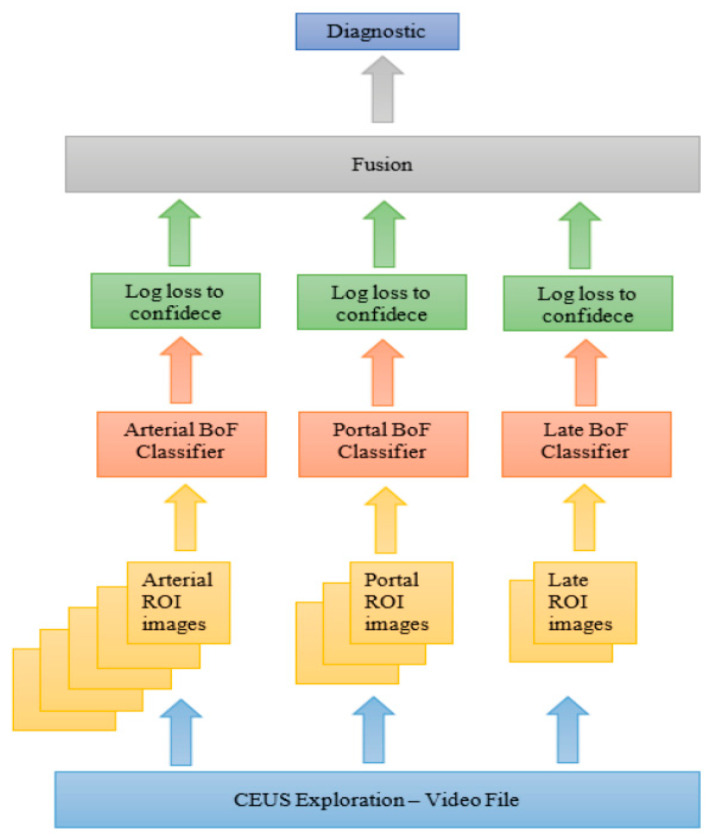
BoF based CAD system for CEUS FLLs diagnosis.

We further proposed an extension in [35] to include spatial information with no need for hand-crafted features. The key aspect was to use a bag of feature (BoF) method in which point selection is performed using a fixed 4 × 4 pixels grid, and SURF descriptors are extracted from 32-, 64-, 96-, and 128-pixels size square blocks. An image is seen as a histogram of visual words, where the size of vocabulary is equal to 400. For each phase of CEUS investigation we trained a BoF based classifier (see Figure 3).

The overall accuracy estimated was 64%. The dataset used had 55 CEUS video files of the following five liver lesions: FNH—11 cases, HCC—11 cases, HMG—11 cases, HYPERM—11 cases and HYPOM—11 cases. For each patient we extract 10 ROI images: five for arterial, three for portal venous and two for the late phase, resulting in a total number of 550 images.

As disadvantages, one could mention long training process, many hyperparameters to optimize and a low accuracy. In order to mitigate the abovementioned disadvantages, we introduce in [10] a 2D-DCNN for implementing an automated diagnosis system which discriminates between an increased number of focal liver lesion types. We proposed a shallow architecture with three convolutional layers, that was trained using ADAM optimizer for 50 epochs, using a 32-batch size, with input dimension 180 × 180, which reached an accuracy of 95.71% using 80% of the available pictures for training and 20% for testing phase. The dataset used in this work has 95 CEUS investigations with the following five liver lesions: FNH—17 cases, HCC—33 cases, HMG—23 cases, hypervascular metastases (HYPERM)—11 cases and hypovascular metastases (HYPOM)—11.

The main drawback of the latter approach is related to the dataset used for evaluation: the images from the training and testing sets, obviously distinct, may still origin from the same patient, thus making the evaluation easier and not suitable for claiming CAD in-field performances.

In the current approach we introduce a new, more realistic, evaluation procedure, referred to as leave-one-patient-out (LOPO). Assuming that the total number of available dataset patients is *N*, a patient-specific leave-one-out *N*-fold cross-validation is used to evaluate the classification accuracy: all images from the same patient are forming the test set whereas the rest of them are used for training purpose. In this way, the reported accuracy is an average obtained over *N* experiments. To our best knowledge, this is the single CAD for CEUS FLLs work in which the evaluation does not follow the standard training/validation/testing split applied with respect to *images*.

The abovementioned procedure enabled us to define and implement different voting schemes for patient-oriented lesion diagnosis. For example, a hard vote scheme predicted class labels for majority rule voting whereas soft voting predicts the class label based on the argmax of the sums of the predicted probabilities.

**Figure 4:**
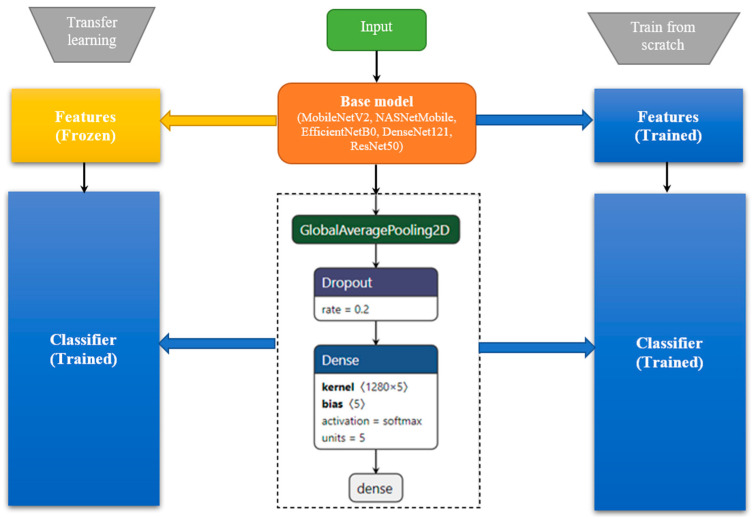
Transfer learning vs. train from scratch paradigm.

The two types of experiments are referring to the same based model used for feature extraction. In transfer learning approach, the layers’ weights are marked as non-trainable whereas in the latter situation they are trained (blue color). In the current work, we further extend [10] by employing modern DNN architectures available through Keras Applications [36]. These are deep learning models that are made available alongside pre-trained weights. In our study, a special emphasis is put on TinyML/small memory footprint models, as we intend to transfer the CAD into a medical embedded system. Figure 4 summarizes the two approaches followed in current study: pre-trained weights/transfer learning vs. train from scratch. Both situations use the same top classifier architecture: global average pooling 2D + dropout + dense layers. The single difference between the two experiments is with respect to the based model used for feature extraction: in transfer learning, the layers’ weights are marked as non-trainable whereas in the latter situation they are trained.

## 4. Results

In this section, we present extensive experimental studies to demonstrate the effectiveness and efficiency of the proposed DL/DNN approach for CEUS FLLs diagnosis.

The experiments were performed using the following setup:

- Hardware architecture: Intel^®^ Core ™ i7—6800K CPU @ 3.4 GHz, 64 GB RAM, 64 bit system, GPU: NVIDIA GeForce RTX 2080 SUPER 1845 MHz, 8 GB RAM, 3072 CUDA Cores
- Software framework: TensorFlow 2.4.1, Python 3.8.5

### 4.1. Custom CNN

**Figure 5:**
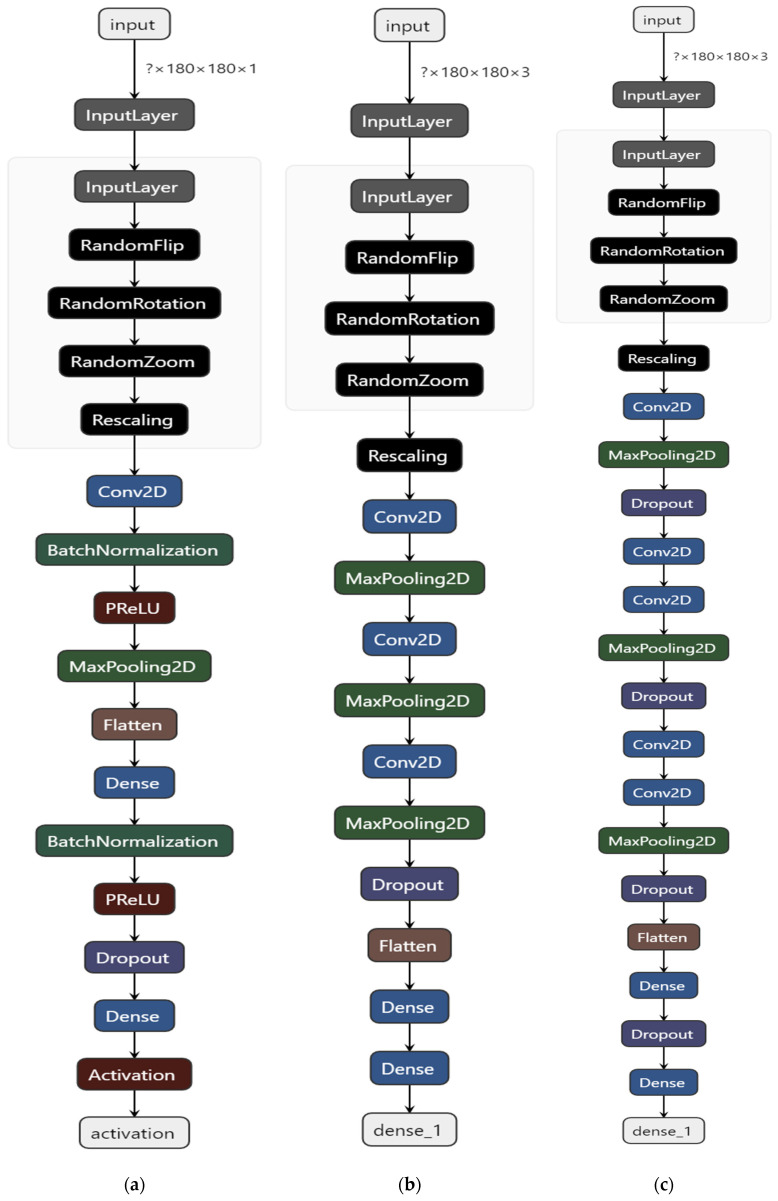
Custom 2D-CNN architectures: (**a**) *Sequential S*—1 convolutional layer; (**b**) *Sequential M*—3 convolutional layers; (**c**) *Sequential L*—5 convolutional layers.

We propose first three empirically designed 2D-CNN models of increasing complexity, namely *Sequential S*—having one convolutional layer; (b) *Sequential M* with three convolutional layers and (c) *Sequential L*—5 convolutional layers. The architectural details are presented in Figure 5.

#### 4.1.1. Evaluation Procedure Influence

Table 2 and Table 3 are presenting the results of a typical 80–20% random train-test split of (possibly) overlapping patient pictures. Next, present the average test accuracy and the corresponding standard deviation under fivefold LOPO cross-validation evaluation procedure. The results with higher accuracy and lower standard deviation are indicated considering, first, an equal number of patients per lesion (Table 4), then, the total number of available patients (Table 5). The best results were highlighted in all below tables.

**Figure 6:**
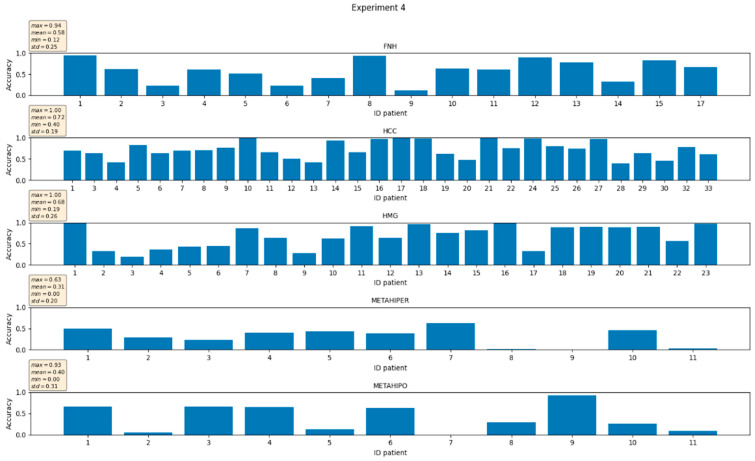
Experiment # 4 from of total of five. Individual accuracies for the case of unbalanced number of examples per class.

**Figure 7:**
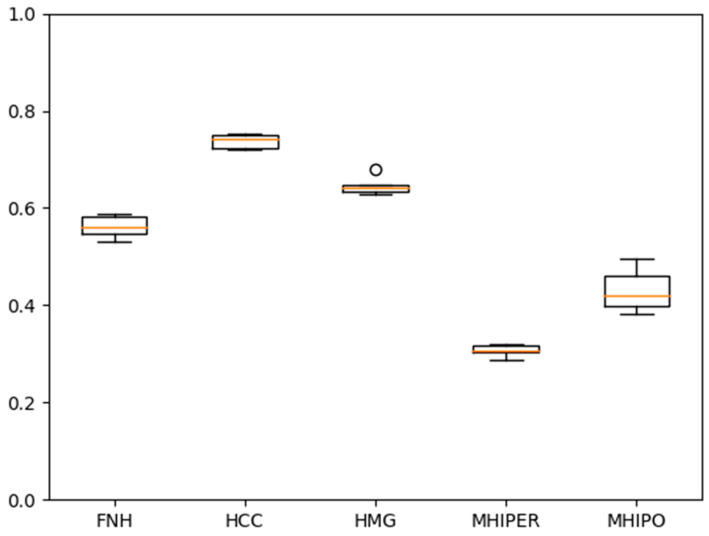
Experiment # 4 from a total of five-accuracies. The Boxplot representation extends from the lower to upper quartile values of the data, with a line at the median.

In Figure 6 and Figure 7, respectively, the individual accuracies for the case of unbalanced number of examples per class of one of the five folds/experiments and, respectively, the average experiment accuracies, are presented.

#### 4.1.2. Voting Scheme

Table 6 and Table 7 present the average test accuracy and the corresponding standard deviation under fivefold LOPO cross-validation evaluation procedure using a *hard vote* scheme: predict the class with the largest sum of votes from the trained model.

**Figure 8:**
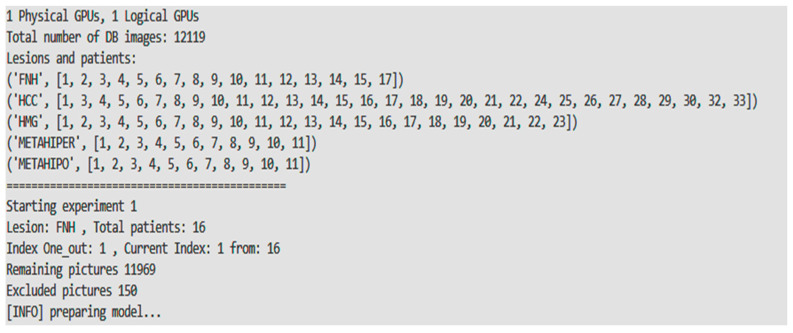
Transfer learning vs. train from scratch paradigm.

For example, in Figure 8, all 150 ROI pictures from FNH patient no. 1 were excluded from the training set. The training procedure will consider just 11,969 ROI pictures, coming from the rest of 90 patients, from the total of 12119. The trained model will predict the correct class label for the test set if at least 1/5th + 1 of the predictions are correct, e.g., [31 FNH, 29 HCC, 30 HMG, 30 METAHIPER, 30 METAHIPO].

**Figure 9:**
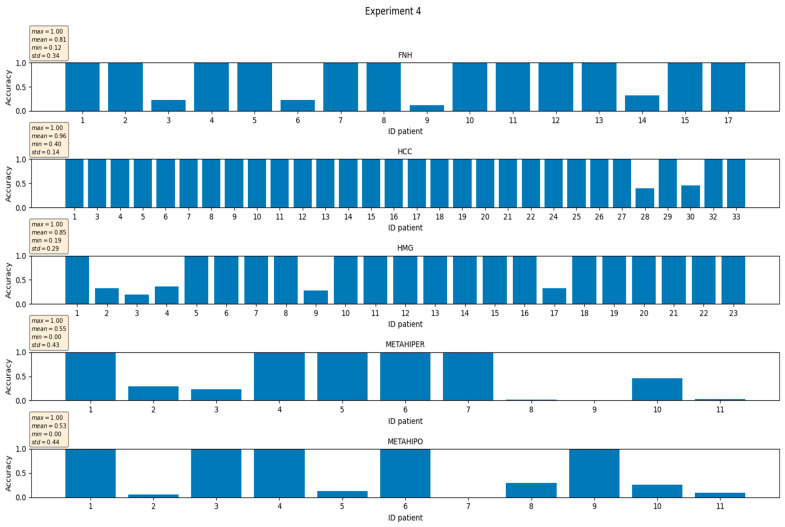
Experiment # 4 from a total of five. Individual accuracies for the case of unbalanced number of examples per class. The hard vote case.

**Figure 10:**
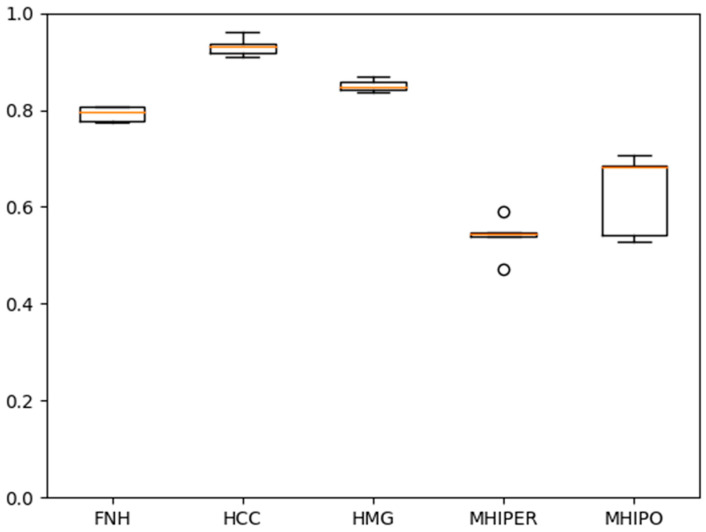
Experiment # 4 from a total of five-accuracies. The Boxplot representation extends from the lower to upper quartile values of the data, with a line at the median. The hard vote case.

In Figure 9 and Figure 10, respectively, the individual accuracies for the case of unbalanced number of examples per class of one of the five folds/experiments and, respectively, the average experiment accuracies, are presented. Hard voting scheme is employed for calculating the decision.

### 4.2. Modern DNN Architectures

In this section, five classic DNN models, available through Keras Applications [36] (deep learning models that are made available alongside pre-trained weights) are evaluated for the CEUS FLLs diagnosis. As could be seen from Table 8, we select the next small size models and compare them with the more complex ResNet architecture:

- MobileNetV2, introduced in [37], has as basic building block a bottleneck depth-separable convolution with residuals; it is faster with the same accuracy than MobileNetV1, and needs 30 percent fewer parameters. Performance on ImageNet showed improvement in state-of-the-art performance points like running time—75 ms, top-1 accuracy—72% or number of multiply-adds—300 M.
- The NASNet [38] research aimed towards searching for an optimal CNN architecture directly on the dataset of interest using reinforcement learning. NASNet Mobile is a simplified version of NASNet which achieves 74% top-1 accuracy, which is 3.1% better than equivalently sized, state-of-the-art models for mobile platforms.
- EfficientNet [39] propose an efficient scaling method that uses a simple yet highly effective compound coefficient. The smallest version of EfficientNet is EfficientNetB0 with a similar architecture to NASNet Mobile which includes a squeeze-and-excite optimization and Swish activation function. The reported top-1 accuracy for EfficientNetB0 is 77.1%.
- DenseNet [40] have several compelling advantages simplifies the connectivity pattern between layers and ensures maximum information flow by connecting every layer directly with each other. It also encourages feature reuse and decrease the number of parameters. It achieved a top-1 accuracy of 75%

To alleviate the difficulty of training a deeper neural network and avoid saturating the accuracy of such networks, ResNet was proposed in [41]. This type of deep CNN has a residual learning layer, where the residual can be simply understood as subtraction of feature learned from input of that layer. ResNet50 is one of the smallest versions, a 50-layer residual network, which has a top-1 accuracy of 75%.

#### 4.2.1. Pre-Trained Modern DNNs

Transfer learning consists of taking features learned on one dataset (usually large, in our case ImageNet), and leveraging them on a new dataset that has less data to train. Although the images in ImageNet are unrelated to medical images, models trained on this dataset can extract more general image features (edges, textures, shapes, etc.).

Transfer learning is usually expressed using pre-trained models. The results are presented in Table 9, using the following data augmentation: randomly flip each image horizontally, rotate and zoom by 10%.

#### 4.2.2. Modern DNNs Trained from Scratch

Here, both base model and the CEUS problem adapted top classifier layers are marked as trainable. The results are presented in Table 10.

## 5. Discussion and Conclusions

The CAD evaluation procedure is a crucial aspect in reflecting the performance. Following the common [11,12,13,14] training/validation/testing split applied with respect to images, by analyzing the effects of the input image size, batch size, training epochs and training algorithm we empirically determine an optimal 2D-CNN architecture (*Sequential M*, Figure 5b) achieving a top 95.71% successful classification for a typical 80%-20% random train-test split of (possibly) overlapping patient pictures (see Table 2 and Table 3). Under newly proposed fivefold LOPO cross-validation evaluation procedure, the accuracy drops to just 56% for the best model (*Sequential S*, Figure 5a). No notable differences between considering all available patients with a specific lesion and the case of equal number of samples per class were observed. The explanation is related to the fact that the fivefold LOPO cross validation is thoroughly and statistical significative. The main disadvantage of the proposed evaluation procedure is that it requires high number of trainings in the loop, 5 (fivefold) × 91 (patients) iterations. These are problematic also due to some memory leaks found in TensorFlow 2.4, solved by clearing memory after each model trains and garbage collection measures.

The earlier results were further improved, by roughly 20%, using the proposed hard voting scheme (Table 6 and Table 7), having *Sequential M* as best performing architecture with a top 75% accuracy. Again, no notable difference between all and just 11 samples per class, when performed fivefold LOPO cross validation were observed.

**Figure 11:**
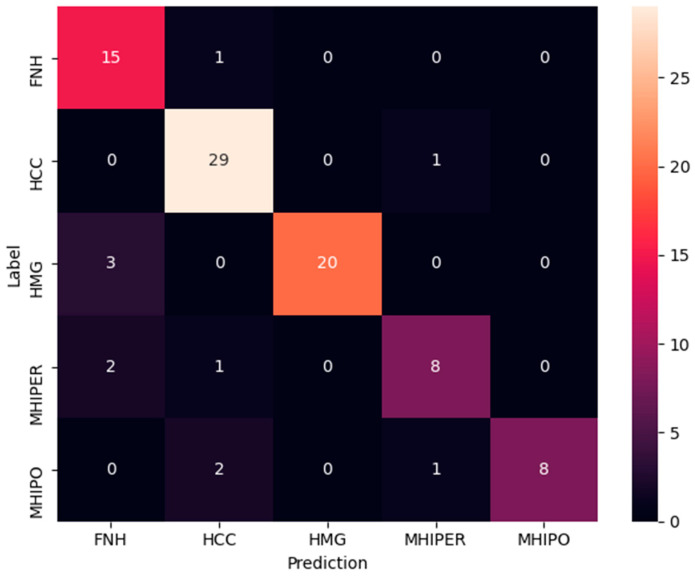
Confusion Matrix for ResNet50V2 trained from scratch.

The last experiment is focused on small footprint modern DNN architecture. The pre-trained/transfer learning approach top accuracy was obtained by DenseNet121, 71% (Table 9). Better results (but with much longer training times) were obtained when training from scratch was employed. The top result was obtained again by the DenseNet121 architecture, with 87% average accuracy. The result is comparable with those obtained using much larger architecture, e.g., ResNet (Table 10) or ResNetV2 (Figure 11), having both 88% average test accuracy.

In this article, steps toward the development of a CAD for CEUS focal liver lesion automated diagnosis using deep neural networks are presented. Custom DNN designs are compared with state-of-the-art architectures, either pre-trained or trained from scratch, using a novel leave-one-patient-out evaluation procedure.

Using a hard voting classification scheme, a top accuracy of 88% was obtained in the automatic diagnosis of five FLL types and both intra- and inter-class imagistic differences are impacting the diagnostic accuracy. In comparison with similar CAD systems, our deep learning-based method provides comparable or better results, for an increased number of FLL types (Table 11).

In future work, it is worth investigating:

- advanced DNN architectures, e.g., GhostNet;
- automatic DNN architecture search, e.g., Autokeras;
- dataset extension, curation—as some cases constantly fail—and enhancement via advanced techniques for speckle noise removal and robustness improvement, e.g., Augmix.

## Figures and Tables

**Table 1:** Number of patients for different FLLs.

FLL	No. of Patients
FNH	16
HCC	30
HMG	23
HYPERM	11
HYPOM	11
**Total:**	**91**

**Table 2:** Optimization algorithm vs. Classification Accuracy [%].

Model	Batch	Training Epochs	Input Size	Adam Optimizer	SGD Optimizer	RMSprop
Sequential M	32	50	180 × 180	**95.71**	93.19	94.59

**Table 3:** Batch, Training Epochs, Input Size vs. Classification Accuracy.

Model	Batch Size	Training Epochs	Input Size	Acc. [%] (Adam Optimizer)
Sequential M	16	50	80 × 80	90.47
Sequential M	16	50	120 × 120	93.93
Sequential M	16	50	180 × 180	91.87
Sequential M	32	50	80 × 80	93.18
Sequential M	32	50	120 × 120	88.77
**Sequential M**	**32**	**50**	**180 × 180**	**95.71**
Sequential M	16	100	80 × 80	94.76
Sequential M	16	100	120 × 120	93.15
Sequential M	16	100	180 × 180	94.72
Sequential M	32	100	80 × 80	92.48
Sequential M	32	100	120 × 120	94.92
Sequential M	32	100	180 × 180	94.43

**Table 4:** Average test accuracy [%] and the corresponding standard deviation. Equal number of examples per class.

Model/Patients	11 FNH	11 HCC	11 HMG	11 MPER	11 MPO	55 Patients
Sequential S	71 ± 0.06	88 ± 0.02	62 ± 0.04	24 ± 0.05	32 ± 0.04	**56**
Sequential M	58 ± 0.05	72 ± 0.07	63 ± 0.08	33 ± 0.04	43 ± 0.04	**54**
Sequential L	45 ± 0.01	88 ± 0.05	60 ± 0.03	14 ± 0.02	36 ± 0.04	**49**

**Table 5:** Average test accuracy [%] and the corresponding standard deviation. Unbalanced number of examples per class.

Model/Patients	16 FNH	30 HCC	23 HMG	11 MPER	11 MPO	91 Patients
Sequentia S	75 ± 0.03	89 ± 0.02	68 ± 0.00	20 ± 0.01	28 ± 0.03	**56**
Sequential M	56 ± 0.02	74 ± 0.01	65 ± 0.02	31 ± 0.01	43 ± 0.04	**54**
Sequential L	49 ± 0.02	84 ± 0.02	63 ± 0.01	15 ± 0.02	33 ± 0.03	**49**

**Table 6:** Average test accuracy [%] and the corresponding standard deviation. Hard Vote and equal number of examples per class.

Model/Patients	11 FNH	11 HCC	11 HMG	11 MPER	11 MPO	55 Patients
Sequential S	86 ± 0.05	99 ± 0.02	77 ± 0.07	31 ± 0.05	46 ± 0.04	**68**
Sequential M	80 ± 0.04	87 ± 0.05	84 ± 0.07	55 ± 0.1	63 ± 0.06	**74**
Sequential L	68 ± 0.05	97 ± 0.02	82 ± 0.04	15 ± 0.04	56 ± 0.05	**64**

**Table 7:** Average test accuracy [%] and the corresponding standard deviation. Hard Vote and unbalanced number of examples per class.

Model/Patients	16 FNH	30 HCC	23 HMG	11 MPER	11 MPO	91 Patients
Sequential S	91 ± 0.04	98 ± 0.02	86 ± 0.03	27 ± 0.02	41 ± 0.03	**69**
Sequential M	79 ± 0.01	93 ± 0.02	85 ± 0.01	54 ± 0.04	53 ± 0.04	**75**
Sequential L	73 ± 0.03	96 ± 0.02	81 ± 0.02	18 ± 0.05	53 ± 0.05	**64**

**Table 8:** Small Size Keras Applications Using the ImageNet Dataset [36].

Model	Size	Top-1 Accuracy	Top-5 Accuracy	Parameters	Depth
MobileNetV2	14 MB	0.713	0.901	3,538,984	88
NASNetMobile	23 MB	0.744	0.919	5,326,716	-
EfficientNetB0	29 MB	-	-	5,330,571	-
DenseNet121	33 MB	0.750	0.923	8,062,504	121
ResNet50	98 MB	0.749	0.921	25,636,712	-

**Table 9:** Average test accuracy [%]. Modern DNN, hard vote, 40 training epochs, early stopping patience 20, equal number of examples per class.

Model/Patients	11 FNH	11 HCC	11 HMG	11 MPER	11 MPO	55 Patients
MobileNetV2	73	94	71	31	71	**68**
NASNetMobile	54	100	76	44	58	**66**
EfficientNetB0	85	94	70	33	54	**68**
DenseNet121	72	100	88	31	62	**71**
ResNet50	69	95	72	43	62	**68**

**Table 10:** Average test accuracy [%]. Modern DNN, hard vote, 40 training epochs, early stopping patience 20, unbalanced number of examples per class.

Model/Patients	16 FNH	30 HCC	23 HMG	11 MPER	11 MPO	91 Patients
MobileNetV2	100	100	93	36	72	**80**
NASNetMobile	100	88	82	68	83	**84**
EfficientNetB0	81	100	90	74	63	**82**
DenseNet121	92	100	94	78	70	**87**
ResNet50	100	100	100	72	67	**88**

**Table 11:** State-of-the-Art Comparison.

Ref.	Lesions	General Accuracy [%]
Hassan et al. [31]	Cyst, HEM, HCC	97.2
Pan et al. [11]	FNH, HCC	93.1
Guo et al. [14]	Malign, Benign	90.4
Vancea et al. [12]	HCC	80.3
Wu et al. [32]	HCC, CH, META, LFS	86.3
Streba et al. [13]	HCC, HYPERM, HYPOM, HEM, FFC	87.1
Ours	FNH, HCC, HMG, HYPERM, HYPOM	88

## Data Availability

The data presented in this study are available on request from the corresponding author of [33]. The data are not publicly available due to copyright.
